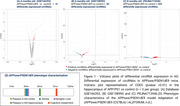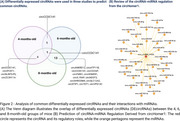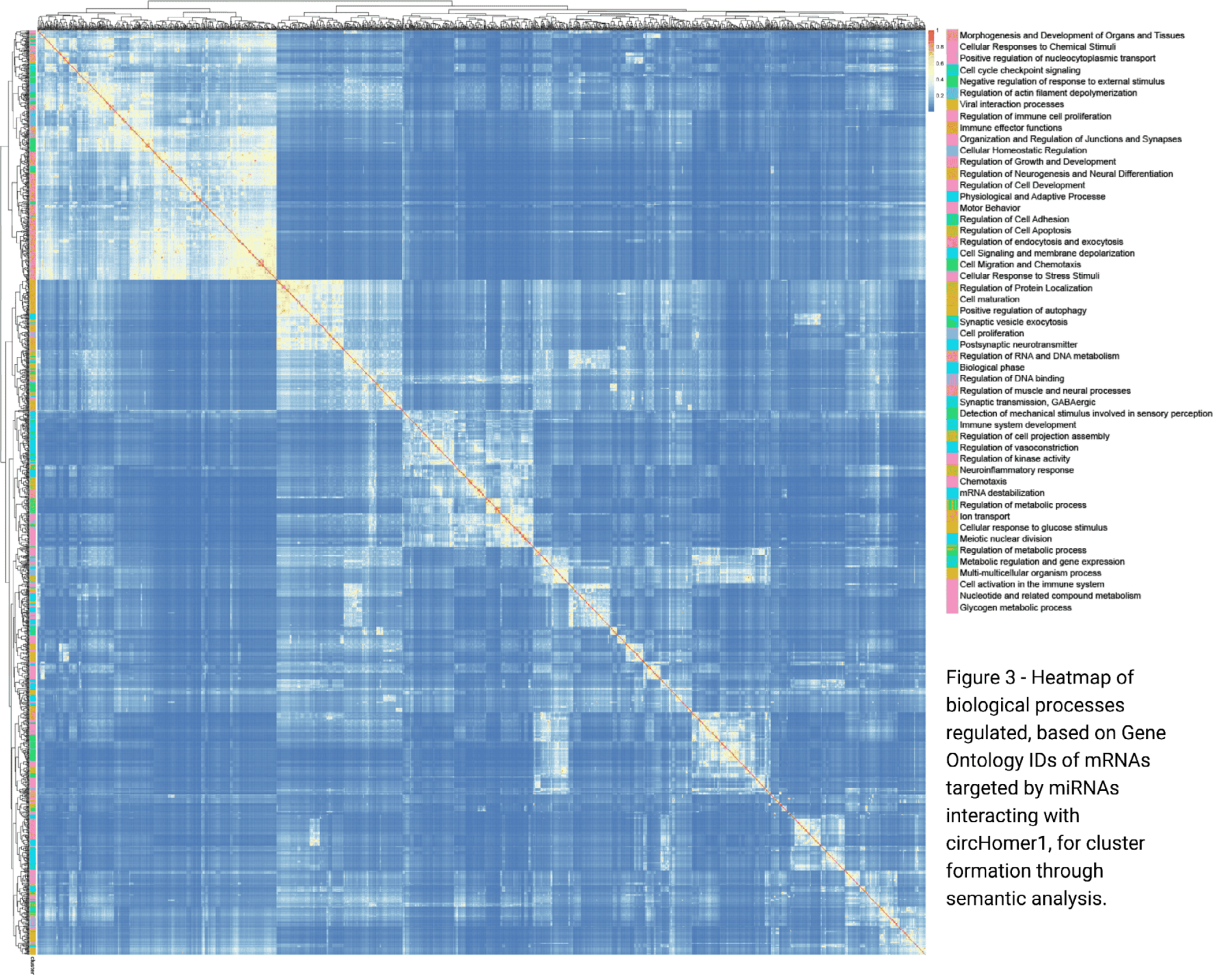# Regulatory Networks of circRNAs, miRNAs, and mRNAs in a APP/PS1 Mouse Model

**DOI:** 10.1002/alz70855_105957

**Published:** 2025-12-24

**Authors:** Lavinia Perquim, Marco Antônio De Bastiani, Oak Hatzimanolis, Alexandre Santos Cristino, Eduardo R. Zimmer

**Affiliations:** ^1^ Universidade Federal do Rio Grande do Sul, Porto Alegre, Rio Grande do Sul, Brazil; ^2^ Griffith University, Queensland, QLD, Australia; ^3^ Griffith Institute for Drug Discovery, Griffith University, Queensland, QLD, Australia; ^4^ McGill Centre for Studies in Agin, Montreal, QC, Canada; ^5^ Brain Institute of Rio Grande Do Sul, PUCRS, Porto Alegre, RS, Brazil; ^6^ McGill Centre for Studies in Aging, Montreal, QC, Canada

## Abstract

**Background:**

Recent advances have highlighted non‐coding RNAs as key regulators of gene expression, including circRNAs, single‐stranded, covalently closed RNAs, and miRNAs, small RNAs that inhibit mRNA translation. Growing evidence suggests that dysregulated expression of both circRNAs and miRNAs is linked to AD. In this study, we assessed whether circRNA transcription is altered in the hippocampus, a region highly vulnerable to AD, and explored the integrated regulation of circRNAs, miRNAs, and mRNAs in a transgenic amyloid mouse model.

**Method:**

We analyzed circRNAs from the hippocampus of the APPswe/PSEN1dE9 mouse model at 4, 6, and 8 months of age, using datasets available in the NCBI database. CircRNA identification was performed with the CIRI2 algorithm, and differential expression was evaluated using DESeq2 (*p*‐value < 0.01) to compare transgenic mice with age‐matched wild‐type controls. Commonly altered circRNAs across studies were identified using Venn diagrams. A regulatory network of circRNAs and miRNAs was constructed with circFunBase, while miRDB was employed to investigate miRNA‐mRNA interactions. Functional enrichment analysis of mRNAs was conducted using Gene Ontology (GO) terms with the ClusterProfiler R package.

**Result:**

We identified 41 differentially expressed circRNAs in 4‐month‐old mice, 82 at 6 months, and 425 at 8 months (Figure 1). The Venn Diagram results showed that a total of 18 circRNAs were shared among the groups (Figure 2A). Of those, we selected circRNA HOMER1, previously described in humans, which interacted with 36 miRNAs (Figure 2B), and these miRNAs, in turn, targeted 1682 genes (Target Score>100). The GO enrichment analysis revealed 51 gene ontologies associated with these genes (Figure 3).

**Conclusion:**

Our results indicate a growing dysregulation of circRNAs in the APPswe/PSEN1dE9, suggesting they follow disease severity. The dysregulation of circRNAs previously described in humans highlights their evolutionary conservation and the advantage of studying them in animal models. Investigating the interactions among circRNAs, miRNAs, and mRNAs revealed genes associated with AD pathophysiology, such as neural differentiation, GABAergic signaling, stress response, and neuroinflammation, suggesting an impact on gene expression in regions vulnerable to the disease.